# Biomarkers for myelin oligodendrocyte glycoprotein-associated disease: recent advances and future

**DOI:** 10.3389/fimmu.2026.1807565

**Published:** 2026-07-31

**Authors:** Qingying Zheng, Muzi Wen, Xinyi Zheng, Linxin Wen, Ruogu Cheng, Ye Gao, Chao Yuan, Pei Shang

**Affiliations:** 1 Department of Neurology, Nanfang Hospital, Southern Medical University, Guangzhou, China; 2 The First School of Clinical Medicine, Nanfang Hospital, Southern Medical University, Guangzhou, China; 3 Department of Neurology, Mayo Clinic, Rochester, MN, United States

**Keywords:** biomarkers, cytokines, inflammatory cell ratios, MOG-IgG antibodies, myelin oligodendrocyte glycoprotein associated disease (MOGAD)

## Abstract

MOGAD is a rare autoimmune encephalomyelitis that shares similar clinical manifestations with other central nervous system demyelinating diseases. However, they must be distinguished due to their distinct pathogenesis and treatment methods. Currently, the clinical diagnosis of MOGAD primarily relies on patients’ clinical manifestations and imaging features, with biomarker application being relatively limited. Meanwhile, research on MOGAD biomarkers has advanced rapidly, with findings mainly focusing on imaging features, antibody titers, nerve cell markers, cytokines, and inflammatory cell ratios. These biomarkers can distinguish among various autoimmune with similar clinical manifestations and exhibit certain specificity. If these biomarker findings are applied to the clinical diagnosis of MOGAD, they could enhance diagnostic accuracy and support earlier and more accurate diagnosis, while providing a scientific basis for evaluating disease progression and prognosis. We discuss the biomarkers and emerging biomarkers of MOGAD, emphasizing the specific changes and aspects that may be applicable to clinical diagnosis and treatment.

## 1 Introduction

Myelin oligodendrocyte glycoprotein-associated disease (MOGAD) is a recently identified rare autoimmune disease characterized by inflammatory demyelination of the CNS in both adults and children (1–3). The core pathogenic mechanism of MOGAD is the “outside-in” model, in which autoimmunity begins in the periphery; disease-associated T cells cross the blood-brain barrier (BBB) during the acute phase, and activated CD4+ T cells open the BBB, allowing MOG-IgG antibodies, complement, and other immune cells to enter the central nervous system (CNS). T cells are re-presented with MOG by antigen-presenting cells (APCs) in the perivascular space or subarachnoid space. Immune cells infiltrate the brain parenchyma, leading to demyelination and inflammatory damage (4, 5). MOG-IgG antibodies are a key pathogenic factor in MOGAD, but they are not the sole causative agent; they require synergy with T cells to induce disease. MOG-IgG binds to MOG on the surface of oligodendrocytes in a bivalent manner, triggering complement activation, ADCC, and enhanced T-cell infiltration into the CNS (6, 7). In terms of complement activation efficiency, MOG-IgG may be more effective than AQP4 IgG at activating C1q; however, the overall degree of complement activation is lower than that observed in AQP4-positive neuromyelitis optica spectrum disorder (NMOSD). In addition, MOG-IgG binding can also induce cytoskeletal disruption in oligodendrocytes (7–9).

The incidence of MOGAD is reported to range between 1.6 and 3.4 cases per million individuals annually, with a prevalence of approximately 20 cases per million (95% CI 11–34). This number is expected to increase as further research into the disease continues (10). Its clinical manifestations resemble those of other CNS demyelinating diseases, including optic neuritis, transverse myelitis, and acute disseminated encephalomyelitis. In the clinical research of treatment options for patients with MOGAD, the diagnostic criteria of MOGAD disease are an extremely important factor, and it is necessary to have an effective diagnostic scheme (11). However, the current clinical diagnosis of MOGAD primarily relies on the clinical manifestations of patients integrated with MRI biomarkers (11–13). This review gives an updated overview of research and development on biomarkers for MOGAD with particular emphasis on their utility in differential diagnosis from other demyelinating diseases of the central nervous system. In recent years, numerous relevant studies have identified useful biomarkers for clinical diagnosis, as well as some emerging biomarkers for the clinical diagnosis of MOGAD.

## 2 Methods

The literature search for this review was conducted in the PubMed/MEDLINE database, with the search period limited to January 2015 through June 2026. This time frame was chosen because, after 2015, the MOG-IgG live cell-based assay (CBA) became the gold standard for diagnosis and MOGAD gained wide recognition as an independent disease entity; following the introduction of the international diagnostic criteria in 2018, research on related biomarkers has increased substantially. A stepwise search strategy was employed to retrieve references. First, relevant literature on MOGAD was identified using the following keyword combinations: “MOG antibody associated disease,” “MOGAD,” “myelin oligodendrocyte glycoprotein antibody-associated disease,” “MOG-IgG,” “anti-MOG,” and “myelin oligodendrocyte glycoprotein antibody.” Subsequently, secondary searches targeting various biomarkers were performed based on these initial results, using the following keyword combinations: (a) MOG-IgG titer: “MOG-IgG titer,” “MOG antibody titer,” and “anti-MOG antibody level” in combination with “relapse,” “prognosis,” or “disease course”; (b) neurofilament light chain (serum neurofilament light chain (sNfL)): “neurofilament light chain,” “NfL,” or “neurofilament light”; (c) tau protein (Tau): “tau protein,” “Tau,” or “total tau”; (d) glial fibrillary acidic protein (GFAP): “GFAP,” “glial fibrillary acidic protein,” or “sGFAP”; (e) cytokines: “cytokine,” “interleukin,” “IL-6,” “TNF-alpha,” “interferon-gamma,” or “chemokine”; (f) microRNAs (miRNAs). Each of the above biomarker searches was combined with MOGAD-related keywords. Finally, a supplementary search targeting core authors in this field was conducted.

References were selected according to the following inclusion criteria: (1) the study population comprised patients with MOGAD meeting the 2018 or 2023 international diagnostic criteria; (2) the study reported quantitative data on at least one biomarker; (3) the study included a control group (healthy controls, multiple sclerosis (MS), or NMOSD or provided longitudinal follow-up data; and (4) the study was a peer-reviewed English-language publication. The exclusion criteria were as follows: studies on MOG-IgG negative demyelinating diseases, conference abstracts, single-case reports, reviews or editorials, publications with incomplete data, and non-English language publications.

## 3 Current diagnostic criteria of MOGAD

MOGAD is a form of autoimmune encephalitis. Studies have shown that the misdiagnosis rate of autoimmune encephalitis remains high, and misdiagnosis of these conditions is a common clinical challenge (12, 14). According to international MOGAD Panel criteria, diagnosis requires:

- Core clinical characteristics of demyelination
- Positive MOG-IgG testing, with supporting clinical or MRI features particularly required for low-positive or unreported titers;
- Exclusion of better alternative diagnosis including MS and NMOSD (10)

The most common clinical manifestations in MOGAD are optic neuritis (ON), myelitis and brain,brainstem, or cerebral syndrome. ON represents the predominant initial manifestation in adults (15–17), while acute disseminated encephalomyelitis (ADEM) is most common in children (18, 19).

Currently, the core challenge in diagnosing MOGAD is that approximately 20% of patients exhibit low or transiently positive antibody tests, and the relationship between antibody titer and disease activity must be dynamically monitored. Due to significant clinical overlap between MS and MOGAD, and NMOSD, MOGAD diagnosis relies heavily on biomarkers to enhance diagnostic accuracy (20–22). Therefore, the search for complementary biomarkers to improve diagnostic specificity, predict disease activity, and monitor treatment response has emerged as a research hotspot. The differential diagnosis of MOGAD is challenging due to partial overlap in clinical phenotypes with two other common central nervous system demyelinating diseases: MS and NMOSD. Given that NMOSD already has a highly specific antibody that enables effective differential diagnosis, this article will focus more on the differential diagnosis between MOGAD and MS rather than NMOSD.

This narrative review summarizes recent advances in MOGAD biomarker research, with emphasis on their potential clinical utility and current limitations, especially for serum and cerebrospinal fluid protein markers, immune inflammation markers, and emerging omics markers. We aimed to provide a directional framework for future directions in translational research.

## 4 Why do we need biomarkers for MOGAD?

MOGAD shares certain clinical similarities with MS, another common central nervous system demyelinating disorders (23). Nevertheless, the prognosis and therapeutic strategies for these two entities differ markedly, necessitating their precise distinction (24–26). Biomarkers for MOGAD would be a key aspect to make this differential diagnosis. The clinical phenotype of MS is predominantly relapsing–remitting (RRMS), with a minority exhibiting primary or secondary progressive courses (27). Disability accumulation is time-dependent; contemporary cohorts show that 30–50% of individuals reach an Expanded Disability Status Scale (EDSS)≥ 6 within 10–15 years of disease onset (28). In contrast to MS, MOGAD often presents with early relapses; nevertheless, the majority of patients experience a favorable long-term outcome without progression to irreversible disability (29). Severe, irreversible disability in MOGAD is uncommon, with cumulative impairment markedly lower than that observed in MS. Long-term cohort data (median follow-up 14 years) demonstrate an EDSS ≥ 6 in only 7% of MOGAD patients, underscoring the condition’s generally benign trajectory (30–32).

## 5 Recent study of biomarkers

At present, there are numerous studies on the biomarkers of MOGAD, but they have not yet been incorporated into clinical practice. These biomarkers can be broadly categorized based on their level of clinical validation. As an established biomarker, MOG-IgG remains the sole serological marker currently used in clinical diagnosis, serving as the cornerstone of MOGAD diagnostic criteria. In the category of exploratory and emerging biomarkers, central nervous system injury-related proteins, including tau protein, NfL, and GFAP, are among the most extensively studied candidate markers in demyelinating disorders, reflecting neuronal damage, axonal injury, and astroglia activation, respectively. Additionally, the specificity of inflammatory cell ratios and changes in multiple cytokines are being actively explored in MOGAD clinical trials.

## 6 Imaging features

Multimodal MRI constitutes a pivotal ancillary tool for diagnosing MOGAD. MOGAD exhibits characteristic MRI signatures in the optic nerves, spinal cord, and brain that facilitate its separation from MS. Optic neuritis in MOGAD demonstrates a predilection for the anterior optic nerve, offering an imaging discriminator. MOGAD-related optic neuritis typically manifests as long-segment T2 hyperintensity confined to the anterior optic nerve, with infrequent chiasmal extension. By contrast, MS lesions may be located anywhere along the nerve, are usually shorter, and are frequently unilateral (33).

A substantial proportion of MOGAD patients present with distinctive spinal cord inflammatory phenotypes. The main characteristic of spinal cord MRI is high signal intensity in the spinal cord gray matter on T2-weighted imaging and demonstrating the long-segment involvement characteristic of neuromyelitis optica, with continuous spinal cord lesions extending over three or more vertebral segments on MRI (34). Unlike MS, MOGAD typically shows a more limited range of spinal cord involvement, with lesions more frequently observed in the lumbar and conus medullaris regions (35, 36). On the sagittal plane, the T2 hyperintense signal band in the spinal gray matter extends in the superior-inferior direction and is surrounded by T2 hyperintensity in the anterior and posterior gray matter horns, forming a distinct H-shaped hyperintensity pattern on the axial plane (37–39). However, on T1-weighted images, MOGAD lesions generally show less persistent T1 hypointensity, suggesting less chronic tissue destruction than is typically observed in MS. This feature aids in distinguishing MOGAD from other demyelinating diseases (40–43).

More than half of patients with MOGAD exhibit brain imaging changes, which are typically bilateral and large-scale and are predominantly localized in the gray matter (10). Some neuroimaging studies have reported cortical and deep gray matter volume reductions in MOGAD, including involvement of temporal, insular, and cingulate regions. Brain gray matter atrophy in MOGAD may be associated with extensive cortical demyelination neuropathologically (16). In virtual histology, gray matter atrophy is inversely correlated with the interregional variation of CA1 and S1 pyramidal cell-specific gene expression (21, 44, 45). It should be noted that in terms of the extent of deep brain gray matter atrophy, MOGAD patients exhibited less atrophy than patients with RRMS, which may aid in differential diagnosis (35, 46, 47). The MRI images of MOGAD patients with partial seizures demonstrate leptomeningeal enhancement, and patients with this feature have a higher prevalence of headache, fever, and epilepsy (48–50).

Some studies have shown that a substantial proportion of MOGAD patients develop mass lesions, a feature that is rarely observed in MS patients. MOGAD patients exhibit hyperintense mass lesions on T2-fluid-attenuated inversion recovery (T2-FLAIR), which may occur in the white matter of bilateral cerebral hemispheres, corticospinal tracts, and thalamus, and may involve the entire pons. However, the frequency of periventricular and other white matter hemispheric lesions was relatively lower (51–53). This lesion feature may be correlated with the severity of clinical manifestations in patients with MOGAD, and patients with tumefactive lesions may require intensive care unit and ventilation treatment (40).

## 7 Titer of antibody

MOG-IgG is an autoantibody targeting human myelin oligodendrocyte glycoprotein. It has been demonstrated that there is a strong correlation between MOG-IgG titers and the clinical manifestations of central nervous system demyelination in MOGAD patients (54–56). Examples include optic neuritis, transverse myelitis, and acute disseminated encephalomyelitis. Among the currently available assays for MOG-IgG detection, the CBA is regarded as the gold standard for the diagnosis of MOGAD. CBA methods for detecting MOG-IgG can be divided into two main types: the live cell-based assay (LCBA) and the fixed cell-based assay (FCBA), and results can be interpreted by microscopy or quantified using fluorescence-based readouts such as flow cytometry. LCBAs using full-length conformational human MOG are generally considered the preferred approach for clinical testing, whereas low-positive results require cautious interpretation in the appropriate clinical context (57, 58). FCBA similarly employs live HEK293 cells expressing MOG; however, following antibody binding, quantitative detection is carried out via flow cytometry, with median fluorescence intensity (MFI) or the FACS ratio (defined as the ratio of MFI in transfected cells to that in un-transfected cells) serving as quantitative readouts. LCBA is simple to perform and requires minimal equipment, rendering it suitable for laboratories with limited resources. Nevertheless, its results are subject to subjective interpretation, which poses challenges for precise dynamic monitoring of antibody titers. In contrast, FCBA, through objective MFI-based quantitative analysis, markedly enhances assay standardization and reproducibility, making it particularly well suited for longitudinal follow up of MOG-IgG titers and multicenter studies. Among patients with CBA-positive results, there was high consistency across different regions, indicating that CBA is a reliable method for detecting MOG-IgG (59–61). High MOG-IgG (≥1:1000) demonstrates higher specificity for MOGAD diagnosis, whereas low (1:20–1:40) carries an increased risk of false-positive results (62).

Detection of MOG-IgG is of crucial importance for the diagnosis of MOGAD. The primary role of this aspect is in distinguishing central nervous system demyelinating diseases with clinical manifestations similar to MOGAD. Several studies have shown that positive MOG-IgG antibodies can serve as a diagnostic prerequisite for MOGAD and have been utilized as a key biomarker in clinical practice. MOG-IgG positive is highly specific for MOGAD (63–65), because the probability of MOG-IgG positive in MS patients is extremely low, thus enabling specific differentiation from MS (26, 66). Owing to the extremely high specificity of MOG-IgG positivity, it has been incorporated into the international diagnostic consensus for MOGAD in the 2023 MOGAD criteria (20). In some cases, MS patients exhibit low-level MOG-IgG positivity, which could potentially mislead clinical diagnosis. Therefore, clinicians should selectively test for MOG-IgG antibodies only in patients with a high clinical suspicion of MOGAD, rather than screening all patients with demyelinating diseases (63).

In addition, it should be noted that in the detection of MOG-IgG in MOGAD patients, MOG-IgG in both serum and cerebrospinal fluid (CSF) was positive in most patients (64, 67–69). Studies indicate that approximately 70% of MOGAD patients exhibit MOGvIgG in both CSF and serum (64). In addition, a few MOG-IgG test results in serum of MOGAD patients are negative, and only CSF test results are positive (65, 68, 69). Approximately 13% of MOGAD patients exhibit serum restricted MOG-IgG positivity, whereas 17% demonstrate cerebrospinal fluid restricted positivity. Cerebrospinal fluid restricted MOG-IgG is associated with ADEM and cortical encephalitis phenotypes within the MOGAD spectrum. This observation suggests intrathecal MOG-IgG synthesis (64). All these MOGAD patients with CSF restricted MOG-IgG meet the diagnostic criteria for MOGAD, and some patients even show high-risk phenotypes of MOGAD (69). Therefore, the positive detection of MOG-IgG in serum should not be the only standard for clinical diagnosis. MOGAD should be diagnosed by clinical symptoms and other auxiliary examination methods.

MOG-IgG also play a significant role in the severity and prognosis of MOGAD. MOG-IgG antibody titers in serum may decline over time or with disease progression (57). Within 6 months, approximately 25% of patients experience a decrease in antibody titers to a low-positive level. Within 12 months, approximately 50% of patients’ antibody titers become negative (58). MOG-IgG titers in most patients with monophasic disease gradually decrease and become negative over several months, whereas patients with relapsing disease may remain MOG-IgG positive. Some patients may even test negative for MOG-IgG between relapses (70). However, if MOG-IgG titers in the tested patient’s serum drop below detectable levels, this may result in false-negative test outcomes (71, 72).

MOG-IgG can play an important role in disease monitoring of MOGAD and may be used to predict whether the disease will follow a monophasic or relapsing course. In monophasic MOGAD, serum MOG-IgG decline over time, whereas in relapsing MOGAD, typically remain elevated (62). Sustained seropositivity for MOG-IgG, particularly at higher, correlates with heightened relapse risk, whereas seroconversion to negativity is linked to a monophasic course and markedly lower relapse risk. MOG-IgG antibodies in MOGAD patients exhibit distinct patterns, including persistent seropositivity, transient seropositivity, and seronegativity. Patients with persistent seropositivity have a significantly higher relapse rate of MOGAD compared to those with transient seropositivity. Persistently positive MOG-IgG antibodies may serve as a reliable predictor of MOGAD relapse (56). However, MOG-IgG should not be regarded as an absolute indicator for detecting MOGAD relapse, given the substantial heterogeneity in MOG-IgG titer kinetics among individual patients (56, 73). Many patients who remain MOG-IgG-positive may still follow a monophasic disease course, whereas a small subset of MOGAD patients may experience relapse even after seroconversion to MOG-IgG negativity (74, 75). This phenomenon further compromises the reliability of persistent MOG-IgG positivity as a predictive biomarker at the individual level. Therefore, further prospective studies are warranted to elucidate the precise role of MOG-IgG in relapse prediction for MOGAD patients.

## 8 Nerve cells biomarkers

Neuronal markers display distinct temporal profiles in MOGAD relative to MS. These neuronal cell markers primarily represent aberrant accumulations of neural proteins secondary to neuronal or axonal injury. Serum measurement of these biomarkers is more accessible and technically robust than CSF analysis. Consequently, this review focuses on serum neuronal-marker findings and their differential specificities versus MS. **Table 1** summarizes the specific manifestations of neuronal cell markers in MOGAD and MS. The combined application of multiple neuronal cell markers may play a significant role in future differential diagnosis and prediction of disease prognosis.

**Table 1:** Nerve cells biomarkers changes in MOGAD.

Nerve cells biomarkers	Biological origin	Reference range (healthy controls)	MS	MOGAD
sNFL	Secondary axonal damage; neuronal cell death	~7.4 pg/mL (median, adults) (87)	Mild to moderate elevation; fluctuates with disease activity (86)	Significantly elevated at first onset (median ~27.2 pg/mL), stabilizes later; positively correlated with (β=1.98, p=0.013) (88)
sTau	neuronal cell; Oligodendrocytes	Not well-established for serum	Mild elevation, more pronounced during progression (4, 97)	Preliminary finding: levels ≥0.37 pg/mL (observed in relapsing MOGAD in a small single-center cohort (n=9) (96); requires validation in larger, multicenter studies) (10)
sGFAP	Mature astrocytes	~61.4 pg/mL (median, adults) (87)	Mild elevation; higher than RRMS during progression (86)	not significantly different between relapse and remission (median ~136.7 pg/mL overall) (154), compatible with spared astrocytes in MOGAD pathology (4)

NMOSD, Neuromyelitis Optica Spectrum Disorder; MOGAD, Myelin Oligodendrocyte Glycoprotein-associated Disease; MS, Multiple sclerosis; NFL, Neurofilament Light Chain; GFAP, Glial Fibrillary Acidic Protein.

### 8.1 NFL protein

**Figure 1:**
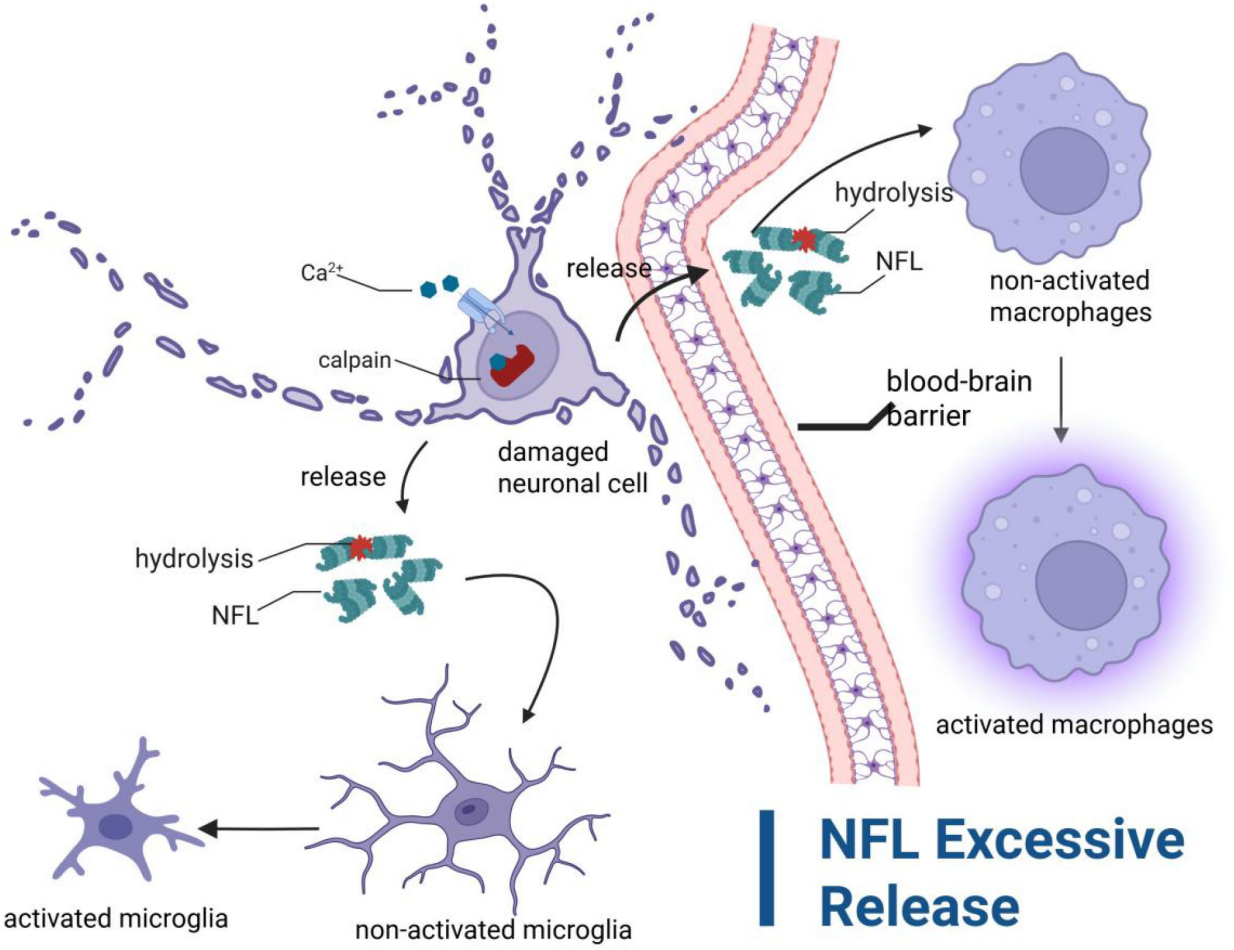
NFL excessive release. When neuronal cells are damaged, neuronal cellular calpain is activated. Calcium ions enter the damaged neuronal cells and bind to calpain, leading to hydrolysis of NFL proteins and the release of large amounts of fragmented NFL. The excessive release of NFL leads to the activation of large numbers of microglia and macrophages. NFL, Neurofilament light chain; Ca, calcium ion.

NFL is a major structural component of neurons (76). In pathological conditions, elevated sNFL levels indicate secondary axonal injury (77). Neuronal damage induces calpain activation, leading to site-specific proteolysis of NFL within the α-helical rod domains and the consequent release of NFL fragments into the bloodstream. The mechanism of excessive NFL release is illustrated in **Figure 1** (78). Excessive NFL release induces microglia and macrophage activation, along with inflammatory responses that may exacerbate the progression of neurodegenerative diseases (79, 80).

Studies indicate that NFL levels in serum and CSF are elevated in MOGAD patients compared to healthy controls (HC). Given that an increasing number of studies have demonstrated that serum NFL levels are more specific than CSF NFL levels and that sample acquisition is more convenient, the focus will subsequently be on serum NFL levels. This difference demonstrates the specificity of sNFL levels in distinguishing MOGAD. Furthermore, sNFL levels are significantly elevated in both adult and pediatric MOGAD patients, suggesting NFL may aid in identifying MOGAD across age groups (81–83). However, these findings are primarily derived from cross-sectional studies with relatively small sample sizes, and the diagnostic thresholds for distinguishing MOGAD from other demyelinating disorders remain undefined. The diagnostic specificity of NFL levels in serum and CSF of MOGAD patients suggests its potential as a reliable biomarker for the disease. Unlike the characteristic of the first onset of MOGAD being elevated and then stabilizing later (84, 85), in patients with MS, sNFL shows mild to moderate increases and fluctuates with disease course (86). This specific change can serve as an important basis for differentiating MOGAD from MS. Nevertheless, the overlap in sNFL levels between MOGAD and MS patients in individual studies limits its standalone diagnostic value, and prospective head-to-head comparisons with standardized assays are lacking. However, it is important to note that NFL concentration increases are nonspecific, as various neurological diseases with axonal injury exhibit varying degrees of NFL elevation. Thus, NFL concentration alone cannot distinguish MOGAD from other neurodegenerative diseases that also show elevated NFL levels. Therefore, the diagnosis of MOGAD necessitates additional clinical evidence or the integration of other biomarkers.

The sNFL level may be used as an important biological marker for the severity and prognosis of MOGAD. NFL concentrations are also positively correlated with patient EDSS scores (87). This suggests NFL level measurement could predict disability and prognosis in MOGAD. Yet, the prognostic value of baseline sNFL levels for long-term disability outcomes has not been prospectively validated, and the correlation coefficients reported across studies vary considerably. sNFL levels are linked to specific clinical symptoms and lesion locations in MOGAD patients. For instance, epileptic seizures are a common clinical feature in MOGAD patients (88). sNFL levels were significantly higher in MOGAD patients with seizures than in those without seizures (81). In MOGAD patients with upper brain lesions, sNFL levels were significantly elevated, as observed in MRI studies (87). These associations, while biologically plausible, are based on retrospective analyses and require confirmation in larger, multicenter cohorts with standardized MRI protocols.

The sNFL levels also serve as a valuable tool in the disease monitoring of MOGAD. The sNFL levels fluctuate during disease progression in MOGAD, peaking during early disease onset or severe attacks (89–91), likely due to new or exacerbated central nervous system inflammation. Thus, a sudden rise in sNFL levels may serve as a biomarker for MOGAD relapse or onset and could assist clinical diagnosis. However, the optimal sampling frequency for monitoring sNFL dynamics and the magnitude of change that constitutes clinically meaningful relapse detection remain undetermined. MOGAD symptom severity correlates directly with NFL levels, possibly because elevated NFL exacerbates central nervous system inflammation, worsening clinical outcomes (92). Overall, while sNFL demonstrates promise as a biomarker for MOGAD diagnosis, severity assessment, and disease monitoring, the current evidence is limited by methodological heterogeneity in assay platforms, lack of standardized cut-off values, and the absence of prospective validation studies. Its clinical applicability at present is best characterized as exploratory, requiring further validation before routine implementation. Moreover, the clinical translation of sNFL is hindered by limited data on inter-laboratory variability, availability of Simoa platforms primarily restricted to specialized centers, cost considerations, and implementation barriers in resource-limited settings, where MOGAD diagnosis may rely on more accessible conventional assays.

### 8.2 Tau protein

Tau protein, a protein expressed in oligodendrocytes, plays an important role in maintaining the physiological function of neurons (93). Under pathological conditions, tau protein gene mutations are associated with neurodegeneration, and elevated levels of abnormal tau protein (e.g., hyperphosphorylation, dephosphorylation, and abnormal tau interactions) contribute to the pathogenesis of neurodegenerative diseases (94). Numerous studies indicate that abnormal tau protein induces disease through two primary mechanisms: pre-tangles and tangles, which impair normal brain neuronal function (95).

The evidence supporting serum tau as a biomarker in MOGAD remains limited, with findings primarily derived from a single research group and requiring independent validation. Serum tau levels can play a significant role in the differential diagnosis between MOGAD and MS. Results demonstrated that serum tau levels in MOGAD patients were elevated compared to the seronegative control group (96). Unlike the elevated tau levels observed during the acute phase of MOGAD, serum tau levels in MS patients exhibit only a mild increase (97). Furthermore, tau levels increase more markedly during the progressive phase of MS compared to the non-progressive phase. Serum tau levels can play a significant role in the disease monitoring of MOGAD. These comparisons are based on relatively small cohorts with heterogeneous control definitions, and the reproducibility of the observed differences across independent centers remains unestablished. Furthermore, serum tau protein levels correlated positively with patient EDSS scores (96). This indicates that serum tau protein levels are positively associated with disease severity and disability extent. The prognostic utility of this correlation for long-term disability prediction has not been prospectively evaluated. Serum tau levels in MOGAD also play an important role in predicting relapses of MOGAD. Longitudinal observational studies indicated higher serum tau levels during relapse than during remission (96). Laboratory data revealed that a serum tau protein level ≥ 0.37 pg/mL is indicative of MOGAD relapse, with test results exhibiting high specificity and sensitivity (96). This proposed threshold originates from a single study with an unspecified sample size for cutoff derivation, and its generalizability across different assay platforms and patient populations is uncertain. Thus, serum tau protein levels may serve as a predictive marker for MOGAD relapse and prognosis. While serum tau demonstrates potential for MOGAD differential diagnosis, severity monitoring, and relapse prediction, the current evidence base is constrained by limited independent replication, methodological heterogeneity in control selection and assay methodologies, and absence of standardized thresholds. Its clinical applicability remains preliminary pending multicenter validation and harmonization of analytical procedures. The clinical implementation of serum tau measurement is constrained by the limited availability of ultrasensitive detection platforms, associated cost considerations, and significant barriers to routine use in resource-limited settings, including low-income countries where access to specialized immunoassays remains restricted.

### 8.3 GFAP protein

GFAP is a key component of the cytoskeleton in mature astrocytes under normal physiological conditions and is widely expressed as a major structural element of the astrocyte cytoskeleton. As widely recognized, astrocytes proliferate extensively in response to nervous system injury and repair processes. Elevated GFAP levels are detectable in various traumatic, ischemic, and neurodegenerative diseases (98–100), making it a potential clinical marker for diagnosing and assessing nervous system damage and repair.

The evidence for sGFAP in MOGAD is currently limited to a small number of cross-sectional studies with modest sample sizes, and its overall diagnostic value remains uncertain. Compared with tau protein and NFL protein, sGFAP exhibits relatively lower specificity in MOGAD patients, as GFAP elevation can occur across multiple neurological conditions involving astroglia activation or injury. This lack of disease specificity represents a fundamental limitation that constrains its standalone diagnostic utility. Current experimental studies indicate that sGFAP levels are significantly elevated in MOGAD patients compared to seronegative controls (87). The control groups in these studies were heterogeneous, comprising both healthy individuals and patients with other neurological disorders, which complicates the interpretation of observed differences. However, sGFAP levels show no significant correlation with EDSS scores (96, 101), indicating that sGFAP cannot predict patient prognosis or assist in assessing the degree of disability. The reproducibility of this negative finding across independent cohorts is uncertain, as only two studies have examined this association to date. Furthermore, sGFAP levels do not differ significantly between MOGAD patients during relapse and remission, limiting its utility in detecting disease onset and progression. sGFAP levels in MOGAD patients are modestly elevated during the acute stage, potentially linked to reactive gliosis occurring in this phase (47, 82). The magnitude of this elevation appears modest relative to other astroglial pathologies, and whether this reflects true disease-related astrocyte activation or non-specific stress responses remains unclear. Additionally, patient age shows no significant association with sGFAP levels (96). sGFAP demonstrates limited potential as a standalone biomarker for MOGAD due to its poor disease specificity, inconsistent association with clinical outcomes, and lack of dynamic responsiveness to disease activity. Its current clinical applicability is restricted, and future studies employing standardized assay platforms and larger prospective cohorts are needed to clarify whether sGFAP may hold value as a complementary biomarker in combination with more specific markers such as sNFL or MOG-IgG titers. The clinical translation of sGFAP is hindered by limited data on inter-laboratory variability, restricted availability of standardized assays outside specialized centers, associated cost considerations, and substantial barriers to routine implementation in resource-limited settings, particularly in low-income countries where access to advanced immunoassay platforms remains limited.

## 9 Cytokines and chemokines

MOGAD is distinctly categorized as a separate form of autoimmune encephalitis and exhibits distinct pathogenic mechanisms at the cellular level compared to other autoimmune (102). Cytokines are critical regulators in the immune system, modulating the development, differentiation, and function of immune cells in central immune organs and peripheral tissues, and regulating systemic immune responses. Current research indicates that the roles of various cytokine types and their abnormal levels in autoimmune encephalitis have been extensively studied and recognized. Presently, investigations into cytokine level abnormalities in MOGAD patients have emerged as a research hotspot, with some severe MOGAD cases showing favorable outcomes following cytokine-targeted therapy. Thus, cytokines may hold significant potential in the future clinical diagnosis and management of MOGAD. A brief overview of prior cytokine research is provided here. Unlike the serum antibody titers and protein levels previously described, cytokine changes are predominantly detected in the CSF. Only a few cytokine changes exhibit greater specificity in serum than in CSF. The following section summarizes the acute-phase cytokines characteristic of MOGAD, which may aid in future differentiation between MS and NMOSD. **Table 2** summarizes the acute-phase cytokine changes in MOGAD, MS, and NMOSD.

**Table 2:** Proinflammatory cytokines changes in MOGAD.

Cytokines/chemokines	Body fluids	Interpretation in MOGAD	MOGAD	MS
IL-6	CSF	A large increase in critically ill cases.	Significant increase; decreases in remission (115)	Mild increase or normal; normal in remission (115)
TNF-α	CSF	The abnormal increase of TNF-α level can change the permeability of BBB	Significant increase (103, 127)	Mild increase (155)
IL-10	CSF	IL-10/STAT3 anti-inflammatory pathway activation (118, 119)	Significant increase (118)	Normal or mild decrease (96)
GM-CSF	Serum	CD4+T cell (96)	Significant increase (111)	Normal or mild decrease (134)
IL-1β	Serum		Peak increase within 1 week of onset; followed by rapid decrease (120)	Mild increase(relapse phase) (121–123)

NMOSD, Neuromyelitis Optica Spectrum Disorder; BBB, Blood-brain barrier; MOGAD, Myelin Oligodendrocyte Glycoprotein-associated Disease; MS, Multiple sclerosis.

### 9.1 IL-6 level

The detection and clinical application of IL-6 are extensively utilized (103, 104). Thus, IL-6 may hold significant potential in the diagnosis and management of MOGAD. The evidence supporting IL-6 as a biomarker specifically for MOGAD remains sparse and largely indirect, with most studies focusing on mechanistic roles rather than validated diagnostic or prognostic utility. Mechanistic analysis reveals that CTRP4, an adipokine, can modulate IL-6 signaling by interacting with IL-6 and its receptor, potentially exacerbating MOGAD inflammation through STAT3 pathway inhibition (105). The excessive release of IL-6 may lead to the excessive activation of T helper 17 (Th17) cells, thereby increasing the permeability of the BBB and promoting the inflammatory response in MOGAD (106, 107). These mechanistic insights, while biologically plausible, are derived primarily from *in vitro* and animal models, and their direct relevance to human MOGAD pathophysiology requires further validation. Acute-phase IL-6 changes are more pronounced in CSF than in serum. Thus, the subsequent description pertains to CSF test results. The IL-6 levels in CSF can be utilized for the differential diagnosis between MS and MOGAD. During the acute phase, IL-6 levels in CSF are markedly elevated in MOGAD, whereas they exhibit only a mild increase in MS, with the situation differing here (108). This comparison is based on a limited number of studies with small sample sizes and heterogeneous assay methodologies, and the reproducibility of these findings across independent cohorts has not been established. Moreover, the diagnostic thresholds and specificity of CSF IL-6 for distinguishing MOGAD from other neuroinflammatory conditions remain undefined.

Currently, IL-6 receptor blockers, including tocilizumab (TCZ), have been preliminarily applied in clinical settings to manage refractory and life-threatening MOGAD cases (109–111). Clinical observations of tocilizumab-treated MOGAD patients indicate that IL-6 receptor blockers exert substantial effects across multiple domains, with rapid and potent efficacy in severe MOGAD cases. Tocilizumab treatment is associated with a marked reduction in the annualized relapse rate and significant decreases in disability progression in seropositive patients (112). It is important to distinguish that these therapeutic observations do not directly validate IL-6 as a biomarker; rather, they support the pathogenic relevance of IL-6 signaling. The evidence for IL-6 as a monitoring biomarker independent of its therapeutic target status remains limited, and whether IL-6 levels can reliably guide treatment decisions or predict response to tocilizumab has not been prospectively tested.

The IL-6 levels in CSF can play a significant role in the disease monitoring of MOGAD. Longitudinal assessments demonstrate that IL-6 levels in CSF are markedly elevated during acute and relapse phases in MOGAD patients, correlating with disease-related antibody production (113). These findings suggest that elevated IL-6 levels are closely associated with MOGAD pathogenesis and clinical parameters. Furthermore, CSF IL-6 levels were significantly higher than serum levels, indicating that excess IL-6 is primarily produced within the CNS (1, 114, 115). These longitudinal data are derived from observational studies without standardized sampling protocols or harmonized assay platforms, and the methodological heterogeneity in IL-6 detection methods (e.g., ELISA, multiplex assays) limits cross-study comparability. The reproducibility of the observed correlations between CSF IL-6 levels and disease activity across independent centers remains uncertain. While IL-6 demonstrates biological relevance in MOGAD pathogenesis and therapeutic targeting, its current evidence base as a standalone biomarker for diagnosis, prognosis, or disease monitoring is preliminary. The clinical applicability of IL-6 measurement is constrained by the lack of standardized thresholds, limited prospective validation, and absence of data on inter-laboratory variability. Future studies employing unified assay protocols and larger multicenter cohorts are needed to establish whether IL-6 can serve as a reliable biomarker in routine MOGAD management.

### 9.2 IL-10 level

In MOGAD patients, IL-10 level changes in the CSF are more pronounced than those in the serum. Thus, the subsequent description primarily highlights specific IL-10 changes in the CSF. The evidence for CSF IL-10 as a biomarker in MOGAD is limited to a small number of studies with modest sample sizes, and its overall diagnostic utility remains uncertain. During the acute phase of MOGAD, IL-10 levels significantly increase. This is distinct from the moderate increase in NMOSD and normal or slightly decreased levels in MS during the acute phase (102, 116, 117). These comparative findings are derived from single-center studies with heterogeneous control groups and varying assay platforms, limiting cross-study comparability and the reproducibility of the observed differences across independent cohorts. The mechanism underlying the significant CSF IL-10 increase in MOGAD is supported by research indicating that continuous IL-10/STAT3 anti-inflammatory pathway activation drives IL-10 elevation in the CSF (118, 119). While this mechanistic explanation is plausible, it is primarily based on *in vitro* and experimental models, and direct evidence linking IL-10/STAT3 pathway activation to measurable CSF IL-10 elevation in human MOGAD patients remains sparse. The diagnostic thresholds for distinguishing MOGAD from NMOSD and MS based on CSF IL-10 levels have not been established, and the clinical applicability of IL-10 measurement is currently preliminary, pending prospective validation with standardized assay protocols and larger multicenter cohorts.

### 9.3 IL-1β level

Unlike the other two interleukins, IL-1β in CSF is less specific than in serum. This assertion is based on limited comparative data, and the relative diagnostic performance of CSF versus serum IL-1β in MOGAD has not been systematically evaluated. Therefore, the following primarily describes the specific changes and comparisons of serum IL-1β. The evidence for serum IL-1β as a biomarker in MOGAD is currently restricted to a small number of studies with limited sample sizes, and its overall diagnostic value remains uncertain. During the acute phase of MOGAD, IL-1β significantly increases, peaking within 1 week of onset, and then rapidly decreases (120). The reproducibility of this temporal pattern across independent cohorts has not been established, and the optimal sampling window for capturing peak IL-1β levels remains undefined. The timing of IL-1β increase can differentiate MS from MOGAD, as MS shows a mild IL-1β increase during relapses (121–123). These comparative findings are derived from studies with heterogeneous assay methodologies and inconsistent control definitions, and the diagnostic thresholds for distinguishing MOGAD from MS based on IL-1β kinetics have not been validated. The clinical applicability of serum IL-1β measurement is currently preliminary, as its prognostic value for predicting relapse severity or treatment response has not been prospectively evaluated, and standardized assay protocols are lacking. While serum IL-1β demonstrates potential as a dynamic biomarker for MOGAD disease activity, the current evidence base is constrained by methodological heterogeneity, limited independent replication, and absence of validated cut-off values. Future multicenter studies employing harmonized detection platforms and standardized sampling protocols are needed to clarify its clinical utility.

### 9.4 TNF-α level

TNF-α may also play an important role in nervous system inflammation in patients with MOGAD. The evidence supporting TNF-α as a biomarker in MOGAD is currently limited and largely inferential, with most studies focusing on mechanistic roles rather than validated diagnostic or prognostic utility. Detection of TNF-α level changes in MOGAD clinical samples indicates that CSF variations are more pronounced than serum variations. This observation is based on a small number of studies with limited sample sizes, and the comparative diagnostic performance of CSF versus serum TNF-α has not been systematically evaluated. Thus, the subsequent description primarily highlights specific TNF-α changes in CSF. Mechanistically, elevated TNF-α may increase BBB permeability, which has an important impact on the stability of the nervous system and may cause the occurrence and development of nervous system inflammatory diseases (124, 125). The significant increase of TNF-α can promote the surface expression of IL-23R on neuronal cells and affect neuronal apoptosis-related enzyme molecules through signal transduction pathway, thus aggravating the apoptosis of neuronal cells (126). These mechanistic insights are primarily derived from experimental models and *in vitro* studies, and direct evidence linking TNF-α-mediated pathways to measurable biomarker changes in human MOGAD patients remains sparse.

The TNF-α levels can play a crucial role in the differential diagnosis and disease monitoring of MOGAD. The current evidence base for these applications is preliminary. Compared with healthy controls and MS patients, the level of TNF-α in CSF of MOGAD patients was increased (103, 127). These comparative findings are derived from single-center studies with heterogeneous control definitions and varying assay methodologies, limiting cross-study comparability. During the acute phase of MS, TNF-α levels exhibit only a mild increase (128). In some patients during the remission phase, TNF-α levels still exhibit a mild increase (129). The reproducibility of these differential patterns across independent cohorts has not been established, and diagnostic thresholds for distinguishing MOGAD from MS based on TNF-α levels remain undefined. The change of BBB permeability in MOGAD patients can make more inflammatory cells enter the central nervous system, leading to neuronal damage (125, 130). The increased level of TNF-α may be related to the apoptosis of oligodendrocytes, which leads to the aggravation of central nervous system inflammation in patients with MOGAD. While TNF-α demonstrates biological relevance in MOGAD pathogenesis, its current evidence base as a standalone biomarker for diagnosis or disease monitoring is constrained by limited independent replication, methodological heterogeneity in assay platforms and control selection, and absence of standardized thresholds. The clinical applicability of CSF TNF-α measurement remains preliminary, pending prospective validation with harmonized detection protocols and larger multicenter cohorts.

### 9.5 GM-CSF level

GM-CSF (Granulocyte-Macrophage Colony-Stimulating Factor) is primarily produced by T cells, mast cells, endothelial cells, and fibroblasts. It promotes the proliferation of various myeloid cells and activates macrophages and dendritic cells in the central nervous system. These cells secrete substantial amounts of GM-CSF, driving inflammation and demyelination in MOGAD (131, 132). The magnitude of GM-CSF elevation can conveniently distinguish MOGAD from MS, as both MS typically exhibit only a mild increase in GM-CSF (102, 133, 134). These findings are derived from single-center studies with heterogeneous methodologies, and their reproducibility across independent cohorts has not been established. Diagnostic thresholds also remain undefined. From the perspective of the pathological mechanisms of GM-CSF and MOGAD, there is a unique association between GM-CSF and CD4-positive T cells in the peripheral blood of MOGAD patients. When stimulated by MOG peptides, CD4-positive T cells in the peripheral blood form a subset that specifically secretes GM-CSF. These cells secrete substantial amounts of GM-CSF, driving inflammation and demyelination in MOGAD (102, 132). While this mechanism is plausible, it is primarily supported by experimental studies, and direct evidence in human MOGAD patients remains sparse. GM-CSF shows preliminary potential for distinguishing MOGAD from MS, but standardized assays and prospective validation are needed before clinical application.

## 10 Inflammatory cell ratio

Under normal physiological conditions, the white blood cell count in CSF is very low. However, in CNS inflammatory diseases, BBB integrity is compromised, allowing inflammatory cells to infiltrate the CSF.

The interaction between inflammatory cells and neuronal cells may drive the progression of neuroinflammatory conditions (135, 136). Thus, an abnormal increase in CSF inflammatory cell count serves as a reliable biomarker for CNS inflammatory diseases, playing a critical role in early disease identification, detection, and prognosis assessment. The evidence specifically supporting CSF inflammatory cell counts as biomarkers for MOGAD is limited, with most studies being single-center and retrospective. The elevation in neutrophil levels may correlate with elevated pro-inflammatory cytokine levels in CSF during acute CNS inflammatory diseases attacks. Activation of epithelial neutrophil-activating peptide 78 (ENA-78) by pro-inflammatory cytokines results in neutrophil recruitment into the CSF (137). In NMOSD, neutrophil dysfunction is characterized by impaired adhesion, migratory capacity, reactive oxygen species (ROS) production, and degranulation (138). Studies indicate that over half of MOGAD patients exhibit elevated CSF white blood cell (WBC) counts via lumbar puncture, with notable neutrophil increases observed in CSF (139). In pediatric MOGAD cases, CSF WBC counts are also elevated (140). These findings are based on small cohorts with heterogeneous sampling protocols, and the reproducibility of neutrophil elevation patterns across independent centers has not been established. Notably, the proportion of patients with detectable neutrophils is higher during acute MOGAD episodes than during remission (139), suggesting that neutrophil detection in CSF may serve as a promising indicator of acute disease activity. Standardized thresholds for defining clinically significant neutrophilia in MOGAD CSF are lacking.

Owing to the diverse roles of inflammatory cells in disease pathogenesis, CSF inflammatory cell ratios can aid in differentiating CNS inflammatory conditions. In MOGAD patients, CSF inflammatory cell ratios exhibit specificity, serving as a reliable biomarker. This assertion is currently supported by limited evidence. The systemic inflammation index (SIRI), calculated as monocyte count × neutrophil-to-lymphocyte ratio (NLR), integrates neutrophil, lymphocyte, and platelet data to assess systemic inflammation. Statistical analyses indicate that the NLR is elevated in MOGAD patients compared to NMOSD patients and healthy controls, with high specificity (140). The optimal sSIRI threshold for MOGAD is 1.565 (140). This threshold was derived from a single study with an unspecified derivation cohort size, and its generalizability across different populations and assay platforms remains uncertain. This finding exhibits high specificity among central nervous system demyelinating diseases and may serve as a reliable biomarker in future clinical diagnostics. Prospective validation in independent multicenter cohorts is needed. Due to divergent pathogenic mechanisms post-neutrophil injury in MOGAD and other types of central nervous system demyelinating diseases, MLR levels correlate with distinct prognostic outcomes (102). Notably, the monocyte-to-lymphocyte ratio (MLR) is significantly reduced during acute-phase MOGAD compared to remission, suggesting that reduced MLR levels may serve as a biomarker for acute MOGAD onset (140). MOGAD patients with lower MLR levels experience fewer disease relapses (140). The reproducibility of these MLR associations across independent studies has not been evaluated, and the methodological heterogeneity in cell counting techniques limits cross-study comparability. Thus, MLR may represent a clinically valuable predictor of relapse risk in MOGAD. Its current clinical applicability is preliminary, pending standardized flow cytometry protocols and larger validation studies.

Lymphocytes play a critical pathogenic role in MOGAD. In the absence of reactive T cells, antibody positivity alone is insufficient to induce CNS inflammation (141, 142). Different types of lymphocytes exhibit certain specificity in the peripheral blood cell spectrum of MOGAD patients. The proportions of Th2 and Th17 cells in peripheral blood were significantly elevated in MOGAD patients (143). Both Treg cells and Th17 cells play significant roles in the assessment of disease severity and in disease prediction. First-episode patients showing a higher Th17 proportion than those experiencing relapse (143). This finding is based on a single study with limited sample size, and its reproducibility requires confirmation. The proportion of Th17 cells in peripheral blood is associated with myelopathy in MOGAD and is positively correlated with EDSS scores, which may aid in elucidating the pathophysiological mechanisms of MOGAD (144). Higher Treg levels correlate with lower EDSS scores in MOGAD, reflecting reduced disability (143). Notably, the proportion of regulatory T cells (Tregs) in acute-phase MOGAD showed an inverse correlation with disease severity, indicating that elevated Treg levels associate with milder clinical manifestations. These T cell subset analyses are derived from small cohorts with variable gating strategies, and the methodological heterogeneity in flow cytometry protocols limits cross-study comparability. The ratio of Th1 to Th17 cells exhibits specificity in the disease monitoring of MOGAD. Th1 cells play a critical pathological role in demyelinating inflammatory diseases of the CNS. The proportion of Th1 cells in peripheral blood was positively correlated with the annual relapse rate of MOGAD. Th17 cells recruit neutrophils, enhance B cell activity, and drive the release of pro-inflammatory cytokines (145–147). While inflammatory cell profiles demonstrate potential as biomarkers for MOGAD diagnosis and monitoring, the current evidence is constrained by limited independent replication, small sample sizes, and methodological heterogeneity in cell counting and flow cytometry techniques. Standardized protocols and multicenter validation are needed to establish their clinical applicability.

## 11 Serum sEV miRNAS

MicroRNAs (miRNAs), as small non-coding RNAs with a length of roughly 22 nucleotides, are of great significance in the regulation of genes. Serum-derived small extracellular vesicle (sEV) miRNAs are secreted by a variety of cells and enter body fluids and have been used as biomarkers for numerous diseases (148, 149). The evidence for sEV miRNAs in MOGAD is limited to a small number of exploratory studies. Currently, the specific changes in sEV miRNAs in MOGAD are being gradually revealed. In comparative studies of MOGAD with healthy controls or other central nervous system demyelinating diseases, different types of miRNAs have shown significantly increased specificity. Among these, hsa-miR-564, hsa-miR-346, and hsa-miR-873-5p can serve as biomarkers to specifically distinguish MOGAD (150, 151). These findings are derived from single-center studies with small sample sizes, and their reproducibility across independent cohorts has not been established. Additionally, certain miRNAs correlate with EDSS and annual relapse rate (ARR), potentially serving as a reliable basis for predicting MOGAD prognosis in clinical settings (150). These correlations lack prospective validation.It provides a promising new tool for non-invasive diagnosis of MOGAD. The mechanism may involve miRNAs crossing the blood-brain barrier within sEVs, thereby playing a role in central nervous system diseases (152, 153). sEV miRNAs show exploratory potential for MOGAD, but standardized assays and multicenter validation are needed before clinical application.

## 12 Future directions and conclusions

MOGAD represents a distinct neuroinflammatory disorder with unique clinical and pathobiological characteristics. This review highlights the emerging role of multimodal biomarkers in refining diagnosis, predicting disease course, and elucidating underlying mechanisms. Research on MOGAD biomarkers has advanced rapidly, expanding from single MOG-IgG detection to multi-dimensional and multi-omics integrated analysis. Currently, MOG-IgG remains the core of clinically available biomarkers, but its detection standardization and dynamic monitoring value require further enhancement. MOG-IgG seropositivity serves as a cornerstone for diagnosis, yet its fluctuating titers and imperfect correlation with disease activity underscore the need for complementary biomarkers. SNFL and GFAP demonstrate promise as dynamic indicators of acute axonal injury and astroglia activation, respectively, while elevated tau protein levels may reflect neurodegenerative processes in chronic phases. Additionally, dysregulated cytokine profiles (e.g., IL-6) and altered inflammatory cell ratios provide insights into the immunopathogenesis of MOGAD, linking peripheral immune activation to CNS-targeted inflammation. Advanced neuroimaging features—such as longitudinally extensive transverse myelitis, cortical encephalitis, or optic nerve sheath enhancement—further enhance diagnostic specificity and aid in differentiating MOGAD from other demyelinating disorders like MS or NMOSD.

Despite these advances, significant challenges and knowledge gaps remain in translating multimodal biomarkers into routine MOGAD management. Substantial assay variability, particularly between LCBAs and FCBAs for MOG-IgG detection, compromises inter-laboratory comparability and hinders the establishment of universal positivity thresholds. Most published studies are limited by small sample sizes, single-center designs, and heterogeneous patient populations with diverse treatment backgrounds. For example, differences between acute and chronic immunotherapy, or between corticosteroid and intravenous immunoglobulin regimens, may confound biomarker measurements and obscure disease-specific signals. Comorbidities, such as concurrent autoimmune disorders or infectious triggers, are also rarely accounted for and may skew cytokine and inflammatory cell profiles. The generalizability of current findings is further constrained by age-specific biological variation. Pediatric MOGAD often presents with distinct phenotypes, such as acute disseminated encephalomyelitis-like presentations, and may exhibit different biomarker kinetics compared with adult-onset disease. In addition, potential ethnic and regional differences in genetic backgrounds and environmental exposures have not been systematically explored. Although several emerging biomarkers have shown promising associations with disease activity, most remain investigational and have not yet been incorporated into formal diagnostic criteria or validated as surrogate endpoints in prospective trials. This translational gap highlights the need for caution when interpreting their immediate clinical utility. Future research should prioritize rigorous methodological harmonization, including standardized protocols for MOG-IgG testing across platforms, multi-center prospective cohorts with adequate statistical power, and stratified analyses accounting for treatment regimens, comorbid conditions, and age subgroups. Given the high pediatric prevalence of MOGAD, age-adapted normative values and non-invasive sampling strategies should be specifically developed for younger populations. Unlocking the full potential of biomarkers for precision medicine in MOGAD will depend on converging multimodal data serological, CSF, imaging, and multi-omics layers, into a unified, clinically actionable framework, supported by artificial intelligence-driven pattern recognition and validated through independent external cohorts. Through such coordinated efforts biomarker science would move from descriptive association to actionable clinical decision making.

## Glossary

ADEM: Acute disseminated encephalomyelitis
AQPAD: Anti-aquaporin-4-IgG-associated disorders
AQP4: Anti-aquaporin-4;ARR, Annual relapse rate
BBB: Blood-brain barrier
CBA: Cell-based assay
CSF: Cerebrospinal fluid
CNS: Central nervous system
EDSS: Expanded Disability Status Scale
ELISA: Enzyme-linked immunosorbent assay
sEV: Extracellular vesicle
FCBA: Fixed cell-based assay
GFAP: Glial fibrillary acidic protein
HC: Healthy controls
IFN: Interferon-γ
IIF: Indirect Immunofluorescence
IVIG: Intravenous immunoglobulin
LCBA: Live cell-based assay
MOGAD: Myelin Oligodendrocyte Glycoprotein-associated Disease
MOGAD-ON: MOGAD-related optic neuritis
MS: Multiple sclerosis
NFL: Neurofilament light chain
NLR: Neutrophil-to-lymphocyte ratio
NMOSD: Neuromyelitis optica spectrum disorder
miRNAs: MicroRNAs
MLR: monocyte-to-lymphocyte ratio
ON: Optic neuritis
RIPA: Radioimmunoprecipitation assay
ROS: Reactive oxygen species
sGFAP: Serum glial fibrillary acidic protein
sNFL: Serum neurofilament light chain
SIRI: Systemic inflammation index
TCZ: Tocilizumab
Th17: T helper 17
WBC: White blood cell.
